# cPLA_2_α^-/-^ sympathetic neurons exhibit increased membrane excitability and loss of N-Type Ca^2+^ current inhibition by M_1_ muscarinic receptor signaling

**DOI:** 10.1371/journal.pone.0201322

**Published:** 2018-12-17

**Authors:** Liwang Liu, Joseph V. Bonventre, Ann R. Rittenhouse

**Affiliations:** 1 Program in Neuroscience, University of Massachusetts Medical School, Worcester, Massachusetts, United States of America; 2 Department of Physiology, University of Massachusetts Medical School, Worcester, Massachusetts, United States of America; 3 Harvard Institute of Medicine, Harvard Medical School & Brigham and Women's Hospital, Boston, Massachusetts, United States of America; Indiana University School of Medicine, UNITED STATES

## Abstract

Group IVa cytosolic phospholipase A_2_ (cPLA_2_α) mediates GPCR-stimulated arachidonic acid (AA) release from phosphatidylinositol 4,5-bisphosphate (PIP_2_) located in plasma membranes. We previously found in superior cervical ganglion (SCG) neurons that PLA_2_ activity is required for voltage-independent N-type Ca^2+^ (N-) current inhibition by M_1_ muscarinic receptors (M_1_Rs). These findings are at odds with an alternative model, previously observed for M-current inhibition, where PIP_2_ dissociation from channels and subsequent metabolism by phospholipase C suffices for current inhibition. To resolve cPLA_2_α’s importance, we have investigated its role in mediating voltage-independent N-current inhibition (~40%) that follows application of the muscarinic agonist oxotremorine-M (Oxo-M). Preincubation with different cPLA_2_α antagonists or dialyzing cPLA_2_α antibodies into cells minimized N-current inhibition by Oxo-M, whereas antibodies to Ca^2+^-independent PLA_2_ had no effect. Taking a genetic approach, we found that SCG neurons from cPLA_2_α^-/-^ mice exhibited little N-current inhibition by Oxo-M, confirming a role for cPLA_2_α. In contrast, cPLA_2_α antibodies or the absence of cPLA_2_α had no effect on voltage-dependent N-current inhibition by M_2_/M_4_Rs or on M-current inhibition by M_1_Rs. These findings document divergent M_1_R signaling mediating M-current and voltage-independent N-current inhibition. Moreover, these differences suggest that cPLA_2_α acts locally to metabolize PIP_2_ intimately associated with N- but not M-channels. To determine cPLA_2_α’s functional importance more globally, we examined action potential firing of cPLA_2_α^+/+^ and cPLA_2_α^-/-^ SCG neurons, and found decreased latency to first firing and interspike interval resulting in a doubling of firing frequency in cPLA_2_α^-/-^ neurons. These unanticipated findings identify cPLA_2_α as a tonic regulator of neuronal membrane excitability.

## Introduction

Following stimulation of a subset of G-protein coupled receptors (GPCRs), acutely activated group IVa cytosolic phospholipase A_2_ (cPLA_2_α) exhibits high specificity for liberating AA from the sn-2 position of PIP_2_ [1–3]. cPLA_2_α activity promotes acute inflammatory responses and oxidative stress associated with neurological disorders, spinal cord injuries, and stroke [4–9]. Additionally, there is growing interest in understanding links between cPLA_2_α and psychiatric disorders (see Rapoport, 2014[10] for review). cPLA_2_α also participates in cellular processes of neurons critical for normal functioning, such as synaptic plasticity [11–15] and ion channel modulation [16–18].

Previously, we found that bath application of AA mimics the voltage-independent inhibition of whole-cell L- and N-type Ca^2+^ current in superior cervical ganglion (SCG) neurons following stimulation of a M_1_R signaling cascade [19–21], referred to as the slow pathway [22]. Similar results were found for L- and N-current from recombinant Ca_V_1.3 and Ca_V_2.2 channels, respectively [16, 23]. M_1_R signaling stimulates increased release of free AA from SCG neurons implicating the acute activation of a PLA_2_ in the slow pathway following muscarinic stimulation [19]. We tested for this possibility and found that pharmacologically antagonizing PLA_2_ activity during M_1_R stimulation minimized inhibition of both whole-cell L- and N-current in SCG neurons [19–21] and recombinant N-current [23]. These findings support the notion that a particular PLA_2_ may participate in M_1_R-mediated modulation of Ca^2+^ channel activity.

cPLA_2_α may be the PLA_2_ involved in slow pathway modulation of N-type Ca^2+^ (N-) channels. Binding of PIP_2_ to L- and N-channels is necessary for normal channel opening [24, 25]. Rapid decreases in plasma membrane levels of PIP_2_ following activation of a voltage-stimulated phosphatase (VSP), which converts PIP_2_ to PIP, rapamycin-induced translocation of inositol-lipid phosphatases, or M_1_R stimulation [24, 25], decreases channel open probability. cPLA_2_α participation in PIP_2_ metabolism follows an initial series of steps previously described for the slow pathway where M_1_Rs via G_q_ stimulate phospholipase C (PLC) to initiate PIP_2_ metabolism (see [26]). We identified cPLA_2_α as the particular PLA_2_ mediating L-current inhibition using cPLA_2_α antibodies as functional antagonists [19]. Moreover, fatty acid release and L-current inhibition were both lost in SCG neurons lacking cPLA_2_α. These findings suggest that cPLA_2_α may be the PLA_2_ mediating N-current inhibition during M_1_R stimulation by metabolizing PIP_2_ molecules associated with N-channels.

Our hypothesis that cPLA_2_α activity may be required for N-current inhibition by M_1_R signaling is at odds with an alternative model where M_1_R signaling stimulates PLC, which metabolizes PIP_2_ once it dissociates from an N-channel. In this latter model, there is no requirement for cPLA_2_α activity [27, 28]. Consistent with a signaling pathway independent of PLA_2_, normal M_1_R mediated N-current inhibition was observed in the perforated-patch configuration in SCG neurons in the presence of pharmacological antagonism of PLA_2_ activity [24]. These results raise questions concerning a role for PLA_2_ in mediating N-channel modulation. We applied additional pharmacological, biochemical and genetic approaches to determine whether cPLA_2_α mediates N-current as well as L-current inhibition by M_1_R signaling in SCG neurons. We present results which show that cPLA_2_α does serve critical roles in normal neuronal functioning: mediating N-current modulation by M_1_R signaling and more broadly by regulating membrane excitability.

## Materials and methods

### Ethical approval

All protocols were approved by the Institutional Animal Care and Use Committee (IACUC) of the University of Massachusetts Medical School. The IACUC specifically approved animal use for this study, which was carried out in strict accordance with the recommendations in the Guide for the Care and Use of Laboratory Animals of the National Institutes of Health. All efforts were made to minimize animal suffering.

### Acutely dissociated SCG neurons

Acutely dissociated SCG neurons were isolated following CO_2_ exposure and/or decapitation from neonatal 1–3 day-old Sprague-Dawley rats (Charles River Laboratories, Wilmington, MA) following the methods of Liu et al. (2001) and from C57BL/6J X 129/Sv mice lacking cPLA_2_α (10–16 weeks old) created in the Bonventre laboratory [29] were obtained following decapitation using the method of Liu et al. (2006). Briefly, each ganglion was removed from the neck region, cut into several pieces, and then transferred into a 25 cm^2^ culture flask containing 5 ml EBSS, 0.5 mg/ml trypsin (Worthington Biochemicals, Freehold, NJ), 1 mg/ml collagenase D (Roche Applied Science, Indianapolis, IN), 0.1 mg/ml DNaseI (Roche Applied Science), 3.6 g/l glucose and 10 mM HEPES. The SCG pieces were incubated at 34°C in a 5% CO_2_/95% O_2_ gassed, shaking water bath. After 1 hour, cell somata were dissociated from ganglion fragments by trituration. The dissociation was stopped by adding 5 ml of Modified Eagle’s Medium (Gibco, Carlsbad, CA) supplemented with 10% FBS, 4 mM glutamine and 100 IU/ml pen-100 μg/ml streptomycin. Cells were pelleted by centrifuging at 500 × g for 5 minutes. The resulting pellet was resuspended in the supplemented DMEM. Dissociated cells from the equivalent of 1 SCG were plated on poly-_L_-lysine (Sigma) coated glass coverslips and incubated in Falcon 35 mm^2^ dishes at 37°C in a 5% CO_2_ environment. Cells were used within 48 hrs of plating. To study the effects of activation of M_1_Rs on N-channel activity, SCG neurons were preincubated with 500 ng/ml PTX for at least 5 hours, to remove inhibition of N-current by activated M_2_/M_4_ muscarinic receptors coupling to the PTX-sensitive, membrane-delimited pathway [26]. Disabling the membrane-delimited pathway isolates N-current modulation by the M_1_R, PTX-insensitive, slow pathway.

### Electrophysiological methods

Standard whole-cell recording methods were used following the methods of Liu et al [23, 30]. Currents and action potentials (APs) were obtained from SCG neurons, plated on poly-L-lysine coverslips and placed in a glass-bottomed recording chamber holding approximately 25 μl of bath solution. Electrodes were pulled from borosilicate glass capillaries (Drummond Scientific Company, Broomall, PA) and fire-polished to a tip diameter of ~1 μm. The total pipette access resistance ranged from 2.0–2.5 MΩ.

For whole-cell or perforated-patch N-current recordings, cells were clamped to -90 mV and 20 ms test pulses were delivered every 4 sec. N-current amplitude was measured 15 ms after the start of the test pulse. To test for voltage-dependent inhibition, a prepulse protocol was used that alternated every 4 secs between a 200-ms prepulse to +80 mV (+PP) or no prepulse (-PP). After a brief (5-msec) return to -90 mV, the membrane voltage was stepped to +10 mV for 100 msec. M-current was elicited by holding the membrane potential at -20 mV and applying a 500-ms hyperpolarizing pulse to -60 mV every 4 sec. M-current amplitude was measured at -60 mV from the decaying time course of deactivating current as the difference between the average of a 10-ms segment, taken 20–30 ms into the hyperpolarizing step and the average during the the last 50 ms of that step. For current-clamp recordings, APs were generated by injecting 40–200 pA of current for either 400 or 1,000 ms. Both voltage-clamp and current-clamp traces were recorded using an Axopatch 200A amplifier (Molecular Devices), a 1401 *plus* interface and Signal 2.16 software (Cambridge Electronic Design (CED), England). Traces were low-pass filtered at 5 kHz using the amplifier’s Bessel filter, digitized at 20–40 kHz and saved on a personal computer. All recordings were made at room temperature (20–24°C).

### Recording solutions

For perforated-patch and whole-cell recordings, the same bath and internal solutions were used. The bath solution contained (in mM): 135 N-methyl-*D*-glucamine (NMG)-Asp, 10 4-(2-hydroxyethyl)-1-piperazineethane-sulfonic acid (HEPES), 20 barium acetate and 0.0005 tetrodotoxin (TTX; Sigma) (293 mOsM). The L-channel antagonist nimodipine (NMN, 1 μM) was included in the bath to minimize the small amount of L-current present in SCG neurons unless otherwise stated. The pipette solution contained (in mM): 123 Cs-Asp, 10 HEPES, 0.1 1,2-bis (o-Aminophenoxy) ethane-N,N,N’,N’-tetraacetic acid (BAPTA; Sigma), 5 MgCl_2_, 4 ATP (Sigma) and 0.4 GTP (Sigma) (264 mOsM). pH for all solutions was adjusted to 7.5. For perforated patch recordings, a 60 mg/ml stock solution of amphotericin B (Sigma) was made up in dimethyl sulphoxide fresh each day. Amphotericin B was added to an aliquot of pipette solution every 2 hours for a final concentration of 0.1 mg/ml.

For measuring whole-cell M-current, the external solution contained in mM: 160 NaCl, 2.5 KCl, 2 CaCl_2_, 1 MgCl_2_, 10 HEPES, 8 glucose, 0.0005 TTX. The pH was adjusted to 7.5 with NaOH. The internal solution contained in mM: 175 KCl, 5 HEPES, 5 MgCl_2_, 0.1 BAPTA, 4 ATP, 0.4 GTP. The pH was adjusted to 7.5 with KOH.

The external solution for recording APs in the current-clamp mode contained (in mM): 120 NaCl, 3 KCl, 4 MgSO_4_, 1 NaH_2_PO_4_, 25 NaHCO_3_, 2 CaCl_2_ (321 mOsM); the pH was adjusted to 7.5 with NaOH. The pipette solution contained (in mM): 110 K gluconate, 30 KCl, 1 MgSO_4_, 1 CaCl_2_, 0.1 BAPTA, 10 HEPES, 4 ATP and 0.4 GTP (297 mOsM); and the pH adjusted to 7.5 with KOH.

NMN (Sigma), oleyloxyethyl phosphorylcholine (OPC; Calbiochem, La Jolla, CA, or Biomol, Plymouth Meeting, PA) and methyl arachidonoyl fluorophosphonate (MAFP; Biomol) were prepared as stock solutions in 100% ethanol and diluted with bath solution to a final ethanol concentration less than 0.11%. The maximal final concentration of ethanol had no significant effect on whole-cell currents. Stock solutions of Oxo-M (Tocris, Ellisville, MO), ω-agatoxin IVA (ω-Aga IV; Sigma), SNX-482 (Sigma), PTX (List Biological Laboratories, Inc., Campbell, CA) and TTX (Sigma) were made up in double distilled water. cPLA_2_ (Cell Signaling, Beverly, MA) and iPLA_2_ (Upstate, Charlottesville, VA) antibodies were diluted directly into the pipette solution.

### Statistical analysis

Data were analyzed using Patch and Signal (CED), Excel (MicroSoft, Seattle, WA) and Origin (MicroCal, Northampton, MA) programs. Data were expressed as means ± SEM. Statistical significance was determined by either a two-way Student’s *t*-test for two means or a two-tailed paired *t*-test. Data were designated as significant if *p* < 0.05.

## Results

### Antagonizing PLA_2_ activity minimizes N-current inhibition by Oxo-M in perforated-patch recordings

**Fig 1:**
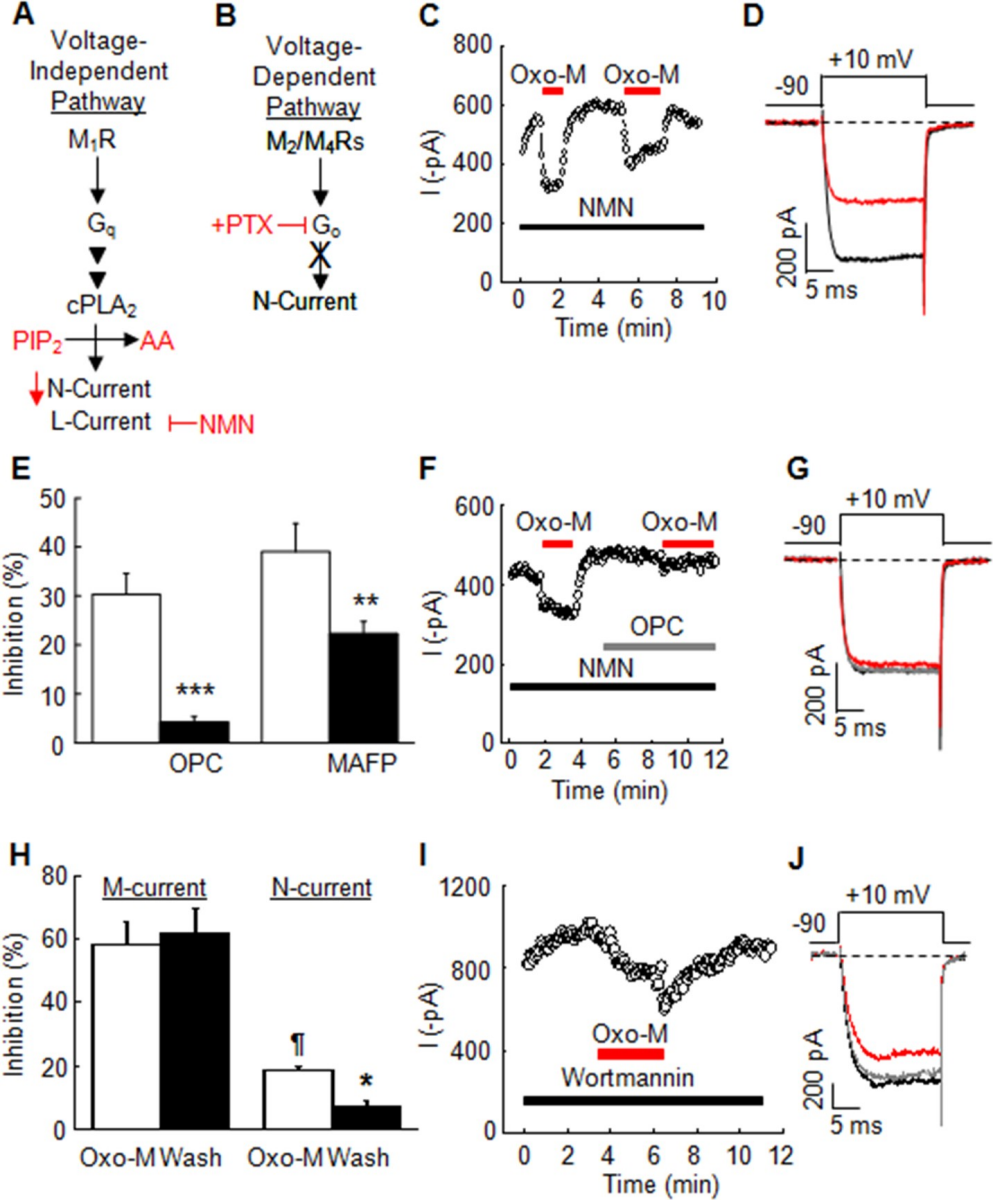
PLA_2_ antagonists reduce N-current inhibition by Oxo-M in neonatal rat SCG neurons. **(A and B)** Schematics showing the two muscarinic signaling pathways in SCG neurons along with the antagonists used that resulted in isolation of N-current by the slow pathway. Control inhibition by 10 μM Oxo-M is illustrated in the plot of current amplitudes vs time **(C)** and in the individual sweeps **(D)** taken from the time course of a perforated-patch recording. NMN (1 μM) was included in the bath to block L-current. **(E)** Summary of effects of PLA_2_ antagonists on N-current inhibition. OPC significantly reduces inhibition of perforated-patch N-current (***p ≤ 0.0015, n = 5) and MAFP (10 μM) significantly reduces inhibition of whole-cell N-current. **p ≤ 0.015, compared to inhibition by Oxo-M alone (open bars) using a two-tailed *t*-test for two means (n = 6–9). The presence of OPC (10 μM) in the bath for 3 min prior to challenging cells with Oxo-M blocks N-current inhibition, illustrated in a sample time course (**F**) and in individual traces (**G**) taken from the time course. (H) Summary of the effects of wortmannin on current inhibition by Oxo-M (open bar) and on current recovery following washout (black bars). M-current amplitude following Oxo-M washout does not recover. N-current inhibition is significantly different than in the absence of wortmannin (^¶^*p* ≤ 0.05, using a two-tailed *t*-test for two means when comparing to data in **Fig 1E**). Significant N-current recovery occurs following washout (*p ≤ 0.03, two-way t-test for two means) (n = 3–9). Example time course (**I**) and individual traces (**J**) of the reversible N-current inhibition by Oxo-M in the presence of wortmannin.

We found previously that treating SCG neurons for two minutes with the PLA_2_ antagonist OPC (10 μM) minimized whole-cell N-current inhibition by Oxo-M [21]. However, Gamper et al. [24] found that, when using the perforated-patch recording configuration, bath application of OPC (10 μM) for two minutes prior to Oxo-M had no effect on N-current inhibition. To determine whether technical differences between the two recording configurations could explain the disparity in results, we tested OPC’s ability to block N-current inhibition in SCG neurons by the slow pathway (**Fig 1A**) using the perforated-patch configuration. Cells were pretreated with PTX to block the membrane-delimited pathway (**Fig 1B**). Under control conditions, Oxo-M (10 μM) reversibly inhibited N-current 30 ± 4.3% [CON = 416 ± 33 pA; Oxo-M = 288 ± 27 pA] (n = 10, *p* < 0.0003) when comparing inhibited to control current amplitudes) (**Fig 1C–1E**). When cells were exposed to OPC for 2 minutes, Oxo-M inhibited N-current by 19% (n = 2), suggesting incomplete antagonism of PLA_2_. Therefore we increased the pre-incubation time to 3 minutes and now found minimal (4.3±1.1%, n = 5) N-current inhibition by Oxo-M (**Fig 1E–1G**), similar to OPC’s actions observed previously in the whole-cell configuration [21]. These results reproduce the findings of Gamper et al [24] and demonstrate that a longer preincubation time with OPC is necessary, when recording in the perforated-patch configuration, in order to observe antagonism of slow pathway modulation of N-current.

### PLA_2_ participates in muscarinic inhibition of whole-cell Ca^2+^ current in cortical neurons

To investigate how widespread is PLA_2_’s involvement in muscarinic modulation of voltage-gated Ca^2+^ channel activity, we tested whether Oxo-M inhibits whole-cell Ca^2+^ currents of prefrontal cortex (PFC) pyramidal neurons (**S1 Methods and S1 Fig**). These neurons receive muscarinic input from the nucleus basalis and express M_1_Rs [31]. Oxo-M inhibited whole-cell currents by 35 ± 11% (n = 2). We then investigated whether antagonizing PLA_2_ minimizes current inhibition and found that in the presence of OPC, Oxo-M no longer inhibited the whole-cell current (-0.3 ± 7.2%; n = 3). These results suggest that PLA_2_ participates in M_1_R-induced Ca^2+^ current inhibition in central neurons as well as peripheral sympathetic neurons. Thus, these OPC experiments are consistent with a role for PLA_2_ in N-current inhibition and that its participation in Ca^2+^ current modulation may be widespread.

### M- and N-current modulation by M_1_Rs exhibit different pharmacological sensitivities

In addition to L- and N-current inhibition by M_1_R signaling, the KCNQ2/3 channel current, M-current, is inhibited by M_1_R signaling via decreased PIP_2_ levels in the membrane by a pathway that appears independent of PLA_2_ [19, 32–34]. Consistent with these findings, we previously found that OPC had no effect on whole-cell native M-current inhibition [19]. Thus, we have used M-current modulation by M_1_R signaling as a negative control for selective N-channel modulation by PLA_2_. In so doing we have identified pharmacological differences in inhibition of these two channels. In addition to a different sensitivity to OPC, a salient feature of M-current is its irreversibility when PIP_2_ resynthesis is blocked by wortmannin in whole-cell and perforated-patch configurations [24, 32]. Wortmannin is a mixed action antagonist that when used at high concentrations (50 μM) inhibits PI4K activity thus limiting synthesis of PIP_2_ [35], but has no effect on basal M-current amplitude or the magnitude of M-current inhibition by Oxo-M.

We compared the effects of wortmannin, on whole-cell M- versus N-current inhibition by Oxo-M to test for further differences in the properties of their respective signaling cascades. When present in the bath solution, 50 μM wortmannin resulted in rapid run down of N-current. However, a lower concentration of wortmannin (10 μM) blocked washout of the muscarinic effect for M-current (**Fig 1H**), similar to what had been observed previously at higher concentrations [32]. Therefore, we tested the effects of this lower concentration of wortmannin on N-current inhibition. We found that the magnitude of N-current inhibition by Oxo-M was significantly less when 10 μM wortmannin was introduced into the bath solution at least 2 min before exposing SCG neurons to Oxo-M (*p* ≤ 0.01; n = 3-7/group). Under these conditions, inhibition reversed upon washout of Oxo-M (**Fig 1H–1J**), similar to wortmannin’s actions on L-current inhibition by Oxo-M [19]. Our findings with a lower wortmannin concentration differ from previous studies of older SCG neurons treated with higher concentrations [24, 36]. Nevertheless, these results are consistent with the possibility that M- and N-current inhibition by M_1_R signaling is mediated by diverging signal transduction cascades.

In addition to differences in the effects of OPC and wortmannin, we found that including BSA in the bath solution blocks N-current inhibition by M_1_R signaling [37], whereas M-current inhibition by Oxo-M remained robust (*p* ≤ 0.00015) in the presence of BSA (445 ± 110 pA in the presence of BSA, 160.9 ± 47.8 pA in the presence of BSA+Oxo-M; n = 7). However, while these experiments suggest differences in signaling, they do not identify whether one key difference is the participation of cPLA_2_α in N-current but not M-current inhibition.

### Identification of cPLA_2_α as the specific PLA_2_ participating in N-current inhibition

Our previous studies demonstrated that L-current inhibition by M_1_Rs specifically required cPLA_2_α, suggesting that it may also mediate N-current modulation [19]. To test this possibility, we examined whether MAFP, an irreversible antagonist that exhibits selectivity for cPLA_2_α [38], blocks N-current inhibition. Preincubation with MAFP (10 μM) for 4 minutes resulted in less N-current inhibition by Oxo-M (22.3 ± 7.5%; n = 9) compared to the control group (29 ± 5.8%, n = 6), consistent with a role for cPLA_2_ in the pathway (**Fig 1E**). However, MAFP alone significantly inhibited N-current by 29 ± 18% (*p* ≤ 0.001; n = 9), raising concern about nonspecific effects on N-channels.

**Fig 2:**
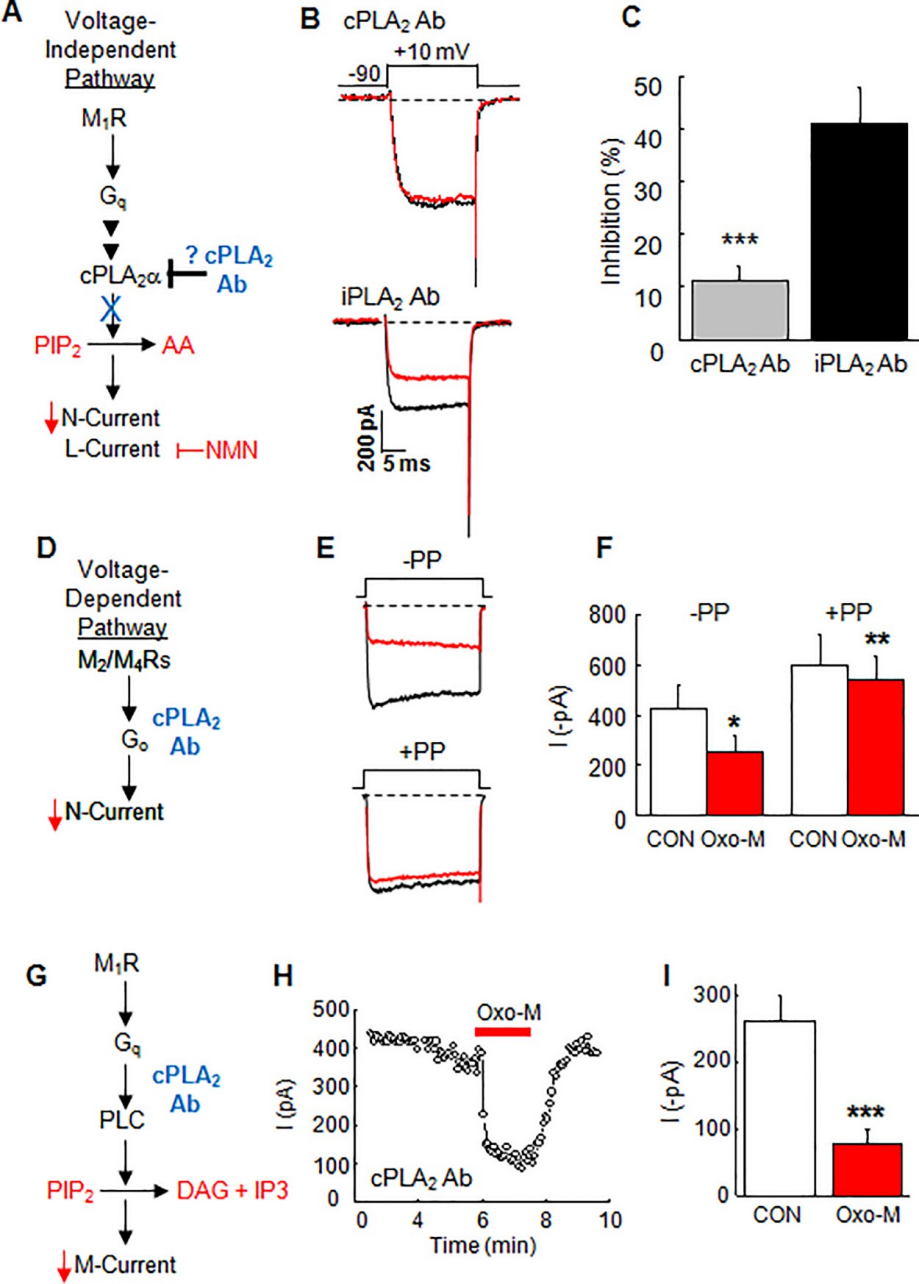
cPLA_2_α is required for inhibition of whole-cell N-current by Oxo-M in SCG neurons. **(A)** Schematic of experimental conditions. (**B**) Individual traces illustrating that dialyzing SCG neurons with cPLA_2_α Abs (Abs, 1:500) minimizes N-current inhibition (n = 8) compared to robust current inhibition (41.2 ± 7.02%) of neurons dialyzed with iPLA_2_ Abs (1:200; n = 4). (**C**) Summary bar graph illustrating the significant difference (***p ≤ 0.001) between the average current inhibition with cPLA_2_α versus iPLA_2_ Abs (n = 4-8/group). (**D**) Schematic of membrane-delimited pathway. (**E**) Individual traces showing N-current inhibition by Oxo-M (red traces) in cells dialyzed with Ab in the absence (-PP) or following (+PP) a prepulse. (**F**) Summary bar graph of control (open bars) and Ox-M inhibited (red bars) N-current amplitude with -PP or +PP protocols. Following Oxo-M, significant inhibition (*p ≤ 0.05; n = 5) occurred in the absence of a prepulse (-PP), which was completely relieved (**p ≤ 0.005; n = 5/group) with a prepulse (+PP). (**G**) Schematic showing the cPLA_2_α Ab should not affect M-current inhibition by Oxo-M. (**H**) Example time course showing rapid, reversible M-current inhibition in SCG neurons dialyzed with Ab. (**I**) Summary bar graph showing significant (***p ≤ 0.0005; n = 8) M-current inhibition by Oxo-M.

Therefore, we took a more specific biochemical approach to identify the PLA_2_ participating in the slow pathway by using selective antibodies as functional antagonists (**Fig 2A**). When an Ab to cPLA_2_α (1:500) was dialyzed into SCG neurons for at least 5 minutes, Oxo-M inhibited N-current 11 ± 2.7% (n = 8; **Fig 2B and 2C**). In contrast, Oxo-M elicited normal N-current inhibition of 41 ± 7.0% (n = 4), when Abs to iPLA_2_ (1:200) were dialyzed into cells (**Fig 2B and 2C**). Basal current levels in cells dialyzed with cPLA_2_α Ab (371 ± 62 pA) compared to those dialyzed with iPLA_2_ Ab (536 ± 156 pA) did not differ significantly (*p* ≤ 0.3), indicating that the antibodies had no effect of their own on N-current amplitude. Moreover, we previously found that dialyzing SCG neurons with IgG also had no effect on N-current inhibition by Oxo-M [20] reporting an average inhibition of 35.4 ± 3.4%.

To examine selectivity for M_1_R-mediated N-current inhibition, we tested whether the cPLA_2_α Ab had any effect on N-current inhibition by M_2_/M_4_Rs. As with the OPC experiments, the Ab experiments were performed with PTX-treated cells. In order to invoke simultaneously both M_2_/M_4_R voltage-dependent and M_1_R voltage-independent N-current inhibition (**Fig 2B**), we continued to use a pipette solution containing a low BAPTA (0.1 mM) concentration, but eliminated neuron preincubation with PTX (**Fig 2D)**. The two forms of inhibition can be distinguished using a prepulse voltage protocol [39] that was initiated immediately following breakthrough (see methods for further details). Under these conditions, a small facilitation of the whole-cell N-current was observed following a prepulse (**Fig 2E and 2F**) most likely due to tonic N-current inhibition by Gβγ [20]. Upon exposure to Oxo-M, N-current was robustly inhibited. Inhibition was partially reversed with a prepulse, revealing the amount of M_2_/M_4_R mediated voltage-dependent inhibition. The remaining voltage-independent N-current inhibition in SCG neurons is known to be mediated by M_1_R signaling [40, 41]. However, when neurons were dialyzed with the cPLA_2_α Ab, prepulses now relieved all of the inhibition by Oxo-M, consistent with inhibition occurring solely via the M_2_/M_4_R mediated voltage-dependent pathway and little to none by the M_1_R voltage-independent pathway (**Fig 2F**). These findings complement the OPC results and are consistent with the cPLA_2_α Ab selectively preventing M_1_R signaling.

Previous OPC experiments suggested that M-current inhibition by M_1_R signaling occurs independently of cPLA_2_α activity [19]. Therefore, we hypothesized that while the cPLA_2_α Ab selectively prevented N-current inhibition by M_1_R signaling, it would have no effect on M-current modulation. We tested this prediction by using the same dialysis protocol as used with N-current modulation (**Fig 2G**). As predicted, robust M-current inhibition by Oxo-M was observed in SCG neurons dialyzed with the cPLA_2_α Ab (**Fig 2H and 2I**). Taken together these studies suggest that M- and N-current are modulated by diverging signal transduction pathways where N-current but not M-current inhibition requires active cPLA_2_α.

### N-channels in cPLA2α^-/-^ SCG neurons are resistant to modulation by the slow pathway

**Fig 3:**
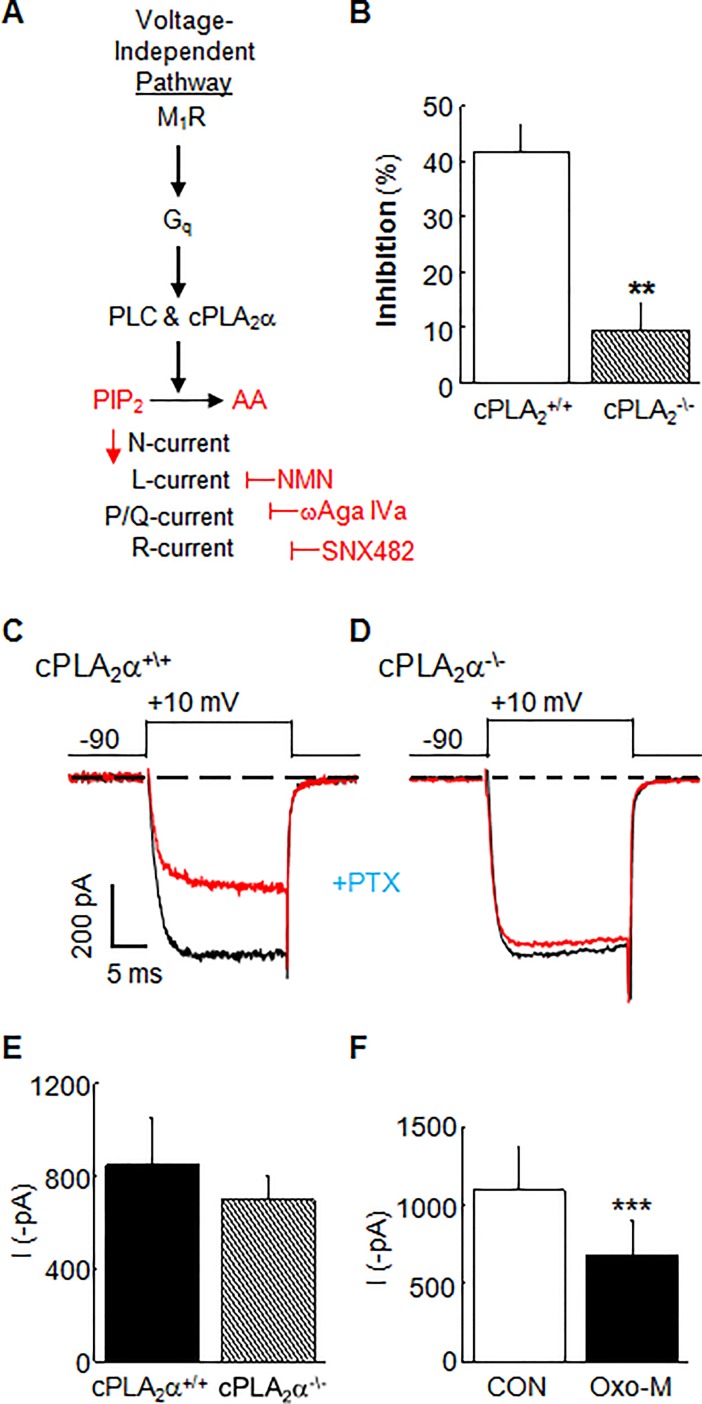
Oxo-M elicits reduced N-current inhibition in cPLA_2_α^-\-^ SCG neurons. **(A)** Schematic of conditions used to isolate N-current in mouse SCG neurons. Cells were preincubated in PTX at least 5 hours prior to recording. **(B)** Summary bar graph of average N-current inhibition by Oxo-M in cPLA_2_α^-\-^ and cPLA_2_α^+\+^ SCG neurons. **p ≤ 0.0015, cPLA_2_α^-\-^ % inhibition compared to cPLA_2_α^+\+^ % inhibition using a two-tailed, *t*-test for two means (n = 5). Individual current traces from **(C)** cPLA_2_α^+\+^ compared to **(D)** cPLA_2_α^-\-^ SCG neurons. Black traces, Control; red traces, Oxo-M. **(E)** Summary of average whole-cell N-current amplitude (NS, *p* ≥ 0.44, n = 5). **(F)** Summary bar graph showing that in the absence of PTX, significant membrane-delimited N-current inhibition by Oxo-M remains in cPLA_2_α^-\-^ SCG neurons remains (***p ≤ 0.00005, n = 10).

Lastly, we took a genetic approach to verify a requirement for cPLA_2_α by testing the effect of Oxo-M on whole-cell N-current in SCG neurons isolated from C57BL/6J x SV-129 mice lacking cPLA_2_α [29]. The cPLA_2_α^+/+^ C57BL/6J and SV129 mouse strains have a naturally occurring background mutation in Group IIa PLA_2_ (sPLA_2_) resulting in a loss of sPLA_2_ activity [42, 43]. Consequently, mice lacking cPLA_2_α are double mutant, but for simplicity are referred to as cPLA_2_α^-/-^ mice. Whole-cell N-currents were recorded from SCG neurons isolated from cPLA_2_α^-/-^ mice (10–16 weeks) and compared to currents from conspecific cPLA_2_α^+/+^ littermates. Mouse SCG neurons express multiple voltage-gated Ca^2+^ channels [19, 44]. Therefore to isolate N-current, a pharmacological strategy previously developed to isolate specific Ca^2+^ currents from mouse SCG neurons was employed [19]. Neurons were pre-incubated with ω-Aga IVa (200 nM) for at least 30 min to irreversibly block any P/Q-type Ca^2+^ current. Recordings were completed within 20 min of the preincubation period. NMN (1 μM) and SNX-482 (20 nM) were added to the bath solution to block L- and R-type Ca^2+^ channels, respectively (**Fig 3A**). Under these conditions, Oxo-M inhibited N-current by 42 ± 4.7% (n = 5) in cPLA_2_α^+/+^ SCG neurons, but only 9.5 ± 4.7% (n = 5) in cPLA_2_α^-/-^ neurons (**Fig 3B–3D**). The magnitude of N-current inhibition by Oxo-M differed significantly between cPLA_2_α^+/+^ and cPLA_2_α^-/-^ SCG neurons (*p* ≤ 0.0015; n = 5). However, unstimulated whole-cell N-current amplitude of cPLA_2_α^+/+^ (851 ± 158 pA) and cPLA_2_α^-/-^ SCG (698 ± 104 pA) neurons did not differ (*p* ≥ 0.35; n = 5 neurons/group), indicating no obvious change in control channel activity in the absence of cPLA_2_α (**Fig 3E**).

**Fig 4:**
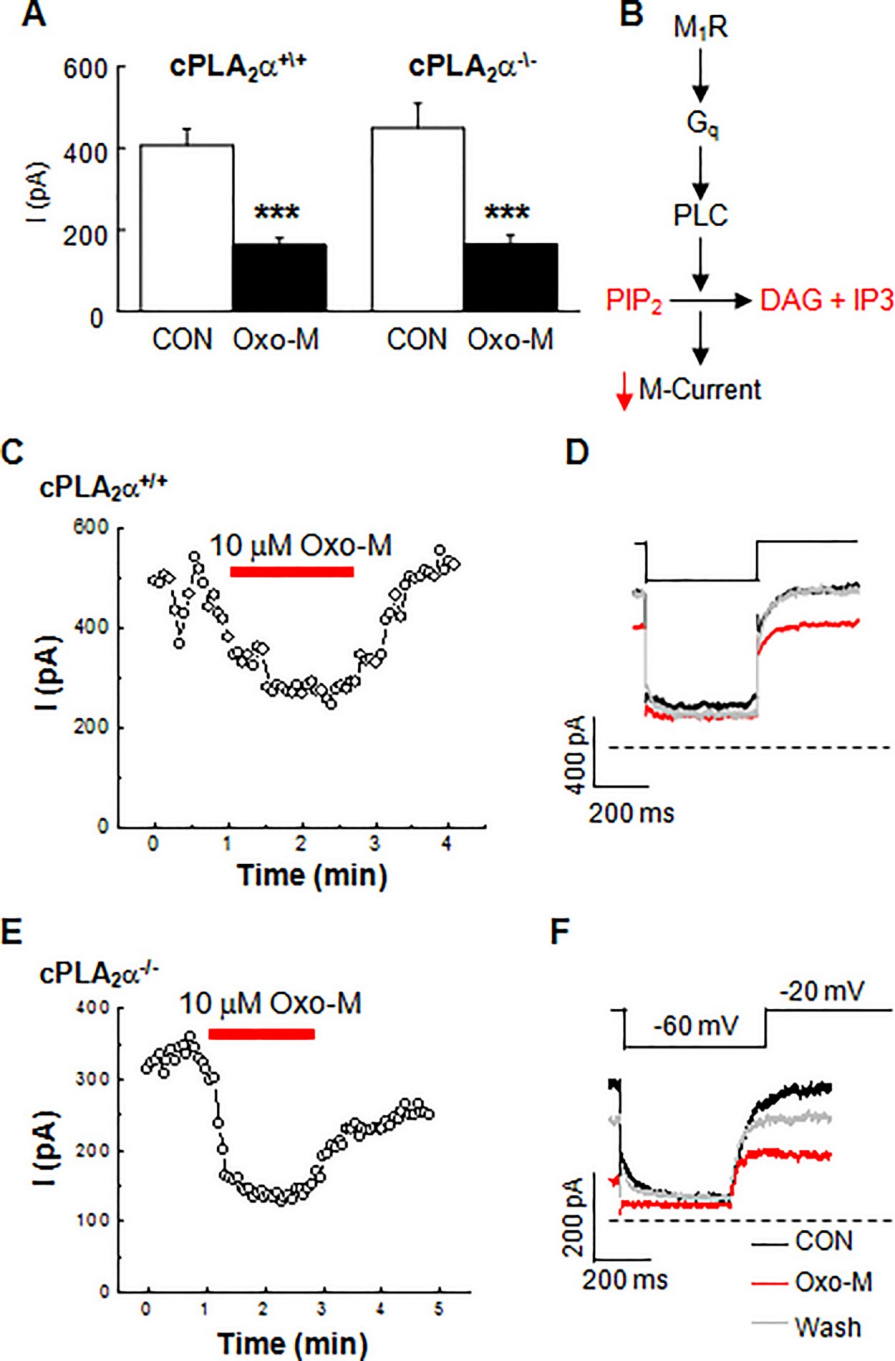
M-current inhibition by Oxo-M remains robust in cPLA_2_α^-\-^ SCG neurons. (**A**) Summary bar graph of the average M-current inhibition in cPLA_2_α^+/+^ (***p ≤ 4 x10^-6^; n = 13) and cPLA_2_α^-/-^ (***p ≤ 6 x 10^−5^; n = 14) neurons following Oxo-M. (**B**) Schematic of the signaling cascade inhibiting M-current. Example time courses and selected individual traces from cPLA_2_α^+/+^ (**C-D**) and cPLA_2_α^-/-^ (**E-F**) neurons.

To test the extent of disrupted muscarinic signaling in the cPLA_2_α^-/-^ SCG neurons, we performed two additional experiments. First, we eliminated the PTX preincubation step and tested cPLA_2_α^-/-^ SCG neurons for N-current inhibition by M_2_/M_4_Rs. Under these conditions, Oxo-M now significantly inhibited N-current by 40.5 ± 4.08% (*p* ≤ 0.00005, n = 10), indicating that the absence of cPLA_2_α had no effect on a different muscarinic signal transduction pathway (**Fig 3F**). Second, we tested for differences in M-current between cPLA_2_α^+/+^ and cPLA_2_α^-/-^ SCG neurons, and found virtually identical average control and inhibited current amplitudes (**Fig 4**) indicating that cPLA_2_α plays no role in regulating basal or modulated channel activity. Additionally, the magnitude of M-current inhibition following Oxo-M of cPLA_2_α^+/+^ (50.6 ± 6.2%) and cPLA_2_α^-/-^ (57.2 ± 3.7%) SCG neurons was not significant (*p* ≥ 0.4; n = 13-14/group). These latter findings provide strong support for a model where M_1_R signaling inhibits N-current by a transduction pathway that requires active cPLA_2_α, by diverging from the pathway mediating M-current inhibition modulation.

### cPLA_2_α regulates action potential firing

**Fig 5:**
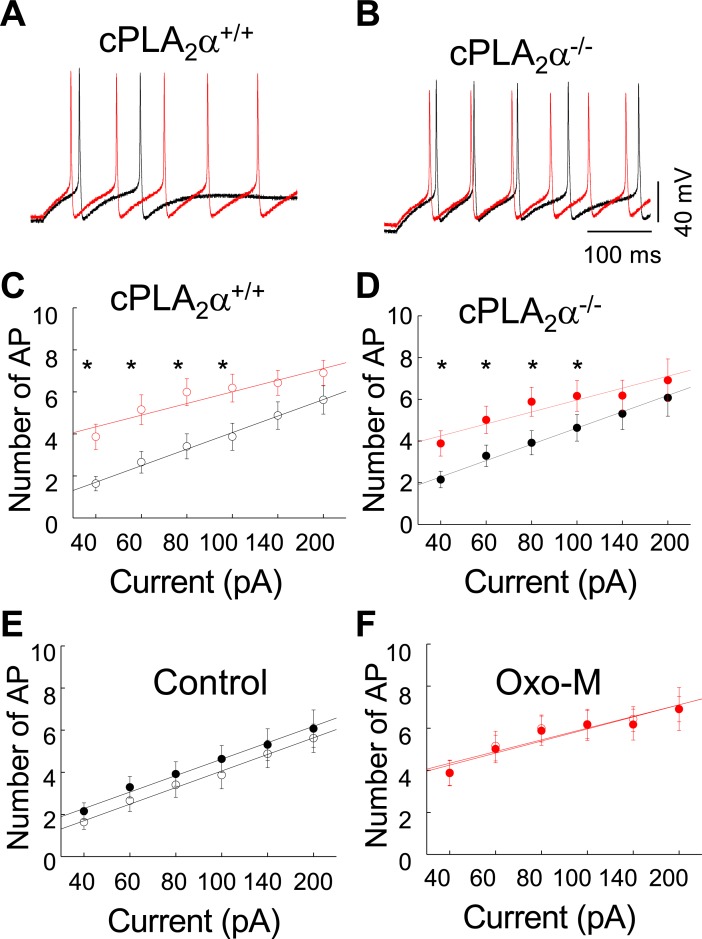
The absence of cPLA_2_α alters AP firing in SCG neurons. (**A-B)** Examples of SCG AP firing elicited with 100 pA. Black traces, control; Red traces, 2 min after Oxo-M. (**C-D)** Current-frequency plots show that firing frequency increases with Oxo-M (red circles). **(E)** Current-frequency plots show that cPLA_2_α^-/-^ neurons (solid circles) exhibit slightly elevated firing frequency over cPLA_2_α^+/+^ neurons (open circles). **(F)** Firing frequency of cPLA_2_α^-/-^ (solid circles) and cPLA_2_α^+/+^ (open circles) cells following Oxo-M.

Many plasma membrane ion channels are affected by interactions with phospholipids and their breakdown products. Therefore, to investigate whether cPLA_2_α^-/-^ SCG neurons exhibit altered electrical properties, we examined the AP firing properties of cPLA_2_α^+/+^ and cPLA_2_α^-/-^ SCG neurons in the absence and presence of Oxo-M. **Fig 5A and 5B** illustrate increased rates of firing in both types of neurons following exposure to Oxo-M. The difference in firing rate was maintained with increasing amounts of injected current. Maximal firing rate occurred with 100 pA of injected current (**Fig 5C and 5D**). While cPLA_2_α^+/+^ and cPLA_2_α^-/-^ neurons did not differ in firing frequency following Oxo-M, cPLA_2_α^-/-^ neurons exhibited unanticipated, increased firing frequency under control conditions when compared to cPLA_2_α^+/+^ neurons that was maintained regardless of the amount of current injected (**Fig 5E and 5F**); however, these increases did not reach significance.

**Fig 6:**
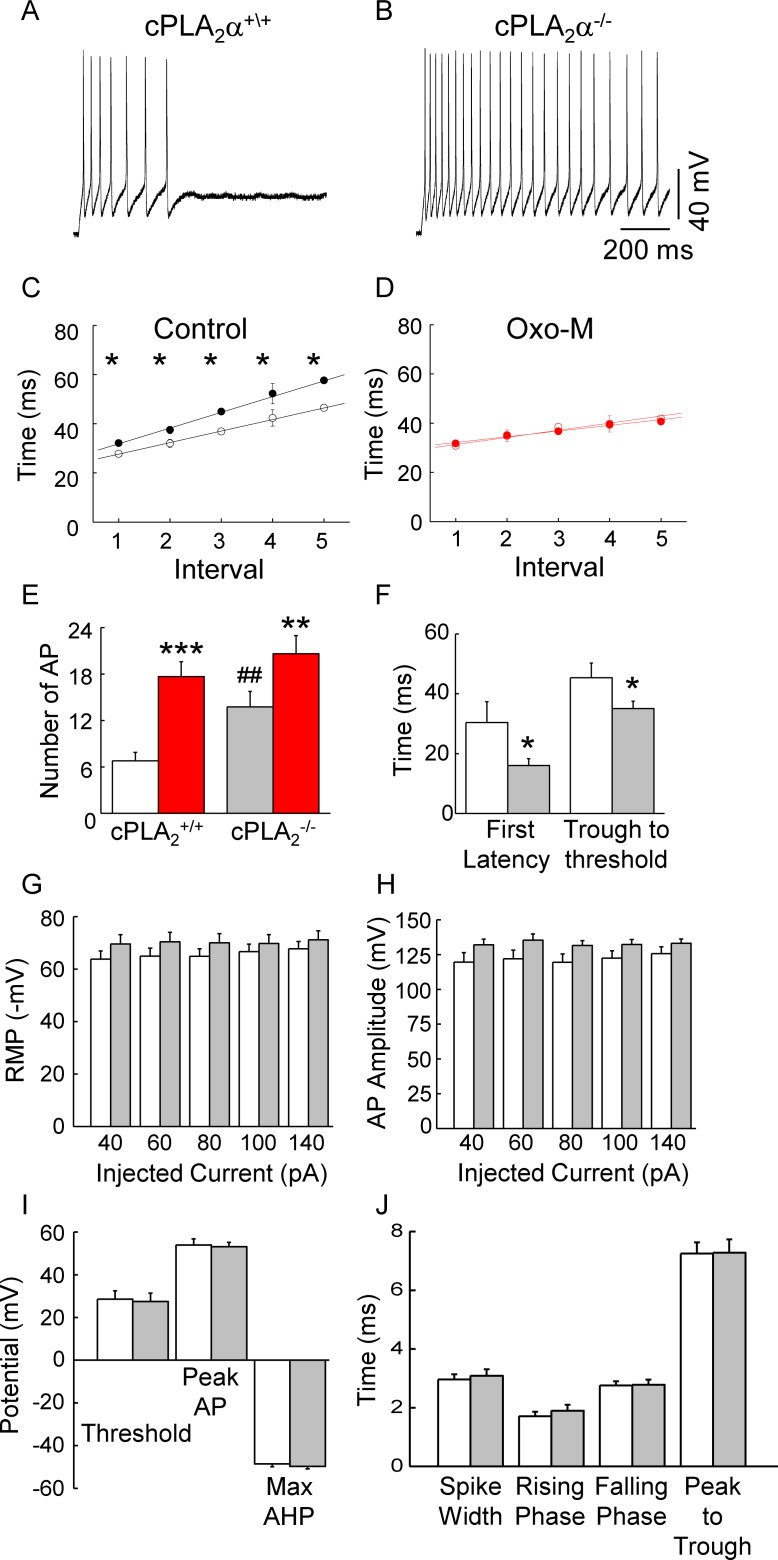
Decreases in time from AHP to threshold account for increased firing of cPLA_2_α^-/-^ SCG neurons. (**A-B)** Examples of differences in AP firing during a 1 sec injection of 100 pA of current. (**C)** IPI is significantly shorter in cPLA_2_α^-/-^ (solid circles) compared to cPLA_2_α^+/+^ (open circles) cells at all intervals (*p ≤ 0.05; n = 17–23 cells/data point). (**D)** Following exposure to Oxo-M, IPI interval lengths of cPLA_2_α^-/-^ (solid circles) and cPLA_2_α^+/+^ (open circles) cells superimpose (n = 8–18 cells/data point). (**E)** cPLA_2_α^-/-^ neurons (n = 22) compared to cPLA_2_α^+/+^ neurons (n = 34) exhibit increased basal (open bars) frequency of firing (^##^*p* ≤ 0.00015), but no difference in frequencies (*p* = 0.377) following Oxo-M (red bars). Both cPLA_2_α^+/+^ (***p ≤ 6 x 10^−5^; n = 18) and cPLA_2_α^-/-^ (**p ≤ 0.005; n = 8) neurons exhibit increases in firing frequency following Oxo-M. **(E-J)** Open bars, cPLA_2_α^+/+^ neurons; grey bars, cPLA_2_α^-/-^ neurons, red bars, 10 μM Oxo-M. All analyses were performed on APs elicited with 100 pA unless otherwise specified. Properties of APs were measured from the 3^rd^ AP of a train from 3 sequential traces and averaged for each recording. (**F)** Decreased latency to first firing (*p ≤ 0.05) and time from the trough of the AHP to threshold (*p ≤ 0.05) underlie the changes in IPI (n = 25–33 cells/group). **(G)** Resting potential and **(H)** AP amplitude of cPLA_2_α^+/+^ (open bars) vs cPLA_2_α^-/-^ (grey bars) SCG neurons were not significantly different (*p* > 0.05; n = 15–18) across a range of current injections. **(I)** Summary of average voltage change from AHP to threshold during an 80 pA current injection (n = 17 cells/group), peak AP (n = 26–27 cells/group), and maximal after hyperpolarization (n = 26–27 cells/group). See **S2 Fig** for a schematic and further details of measurements. **(J)** Summary of the AP duration. Spike Width, measured at ½ the AP amplitude; Rising Phase, measured at 1/3 the AP potential to the peak AP; Falling Phase, measured from the peak AP to 1/3 the AP potential; Peak to Trough, measured from the peak AP to the AHP (n = 26–27 cells/group).

cPLA_2_α^+/+^ SCG neurons normally fired several APs and then adapted despite continued current injection. A small percentage (14%) of cells fired only 1 AP (n = 5/34 recordings). We realized the sampling time in the prior experiment may have been too short to reveal significant differences in firing frequencies. Indeed, when the current injection time was increased from 400 to 1,000 msec, significant differences in basal firing frequency resulted (**Fig 6A and 6B**). 50% of cPLA_2_α^-/-^ neurons (11/22) fired for the duration of the 1,000 msec current injection, whereas 82% of cPLA_2_α^+/+^ neurons (27/33) adapted and ceased firing by 750 msec. We quantitated the underlying changes in the AP waveform (see S2 **Fig**) of phasic and tonic neurons by plotting duration of interpulse interval (IPI) against interval number and found shorter IPIs during control firing for cPLA_2_α^-/-^ compared to cPLA_2_α^+/+^ neurons (**Fig 6C and 6E**). Moreover, the time from the trough of the after hyperpolarization (AHP) to the threshold of the subsequent AP significantly decreased (**Fig 6F**). Consistent with these changes, the latency to the first AP (first latency) occurred in approximately half the time in cPLA_2_α^-/-^ compared to cPLA_2_α^+/+^ neurons (**Fig 6F**). We also observed consistent small increases in both the resting membrane potential (**Fig 6G**) and AP amplitude (**Fig 6H**) with various amounts of current injected; however, these changes did not reach significance. Additionally, no change in the voltage difference between the AHP and threshold of the subsequent AP, peak voltage of the AP overshoot, and AHP amplitude were observed (**Fig 6I**). Lastly, we measured different aspects of the AP duration (**Fig 6J**). We found no differences in spike width, duration of the rising phase of the AP (Rise Phase), duration of the falling phase, measured either from the peak of the AP to 1/3 of the AP’s height (Falling Phase) or from the peak of the AP to the AHP (Peak to Trough). The observed changes resulted in a doubling of basal firing rate for cPLA_2_α^-/-^ compared to cPLA_2_α^+/+^ neurons as measured by the number of APs/second during current injection of 100 pA (**Fig 6E, open vs grey bars**). In contrast, firing frequency of both cPLA_2_α^-/-^ and cPLA_2_α^+/+^ neurons increased to a similar extent following exposure to Oxo-M (**Fig 6D and 6E, red bars**).

## Discussion

This study examined the role of cPLA_2_α in regulating phospholipid association with N-channels in SCG neurons. We took a multidisciplinary approach to test an emerging model of channel modulation where multiple phospholipases including cPLA_2_α act in highly specific ways to metabolize phospholipids such as PIP_2_ at or near N-channels following M_1_R stimulation. Our working model provides a high degree of selective and local control over phospholipid interaction with N-channels. This is in contrast to a model where PLC activity reduces bulk PIP_2_ levels in the cell membrane, which indirectly lowers phospholipid levels near N-channels [24]. Importantly, our data reconcile previous differences surrounding the role of cPLA_2_α in N-channel modulation by G_q_ signaling and reveal a broader role for cPLA_2_α in regulating membrane excitability.

The pharmacological evidence reported here supports a mechanism of modulation where N-current inhibition by M_1_R stimulation requires cPLA_2_α in addition to PLC. Antagonizing cPLA_2_α with OPC reduced N-current inhibition, recorded in the perforated-patch configuration (**Fig 1E and 1F**). We discovered that in order to antagonize N-current inhibition by Oxo-M, a longer pre-incubation period with OPC is required most likely due to a greater maintained intracellular cPLA_2_α concentration with the perforated-patch configuration compared to the whole-cell configuration. The longer preincubation time may allow more OPC to cross the plasma membrane, enter the cell, and inhibit additional cPLA_2_α molecules that remain during perforated-patch recordings. Thus, we suggest that the most likely reason OPC did not prevent N-current modulation in a previous study is due to technical differences between the recording configurations [21, 24]. Additional antagonists of cPLA_2_α (AACOCF3, DEDA, and MAFP) also reduce whole-cell N-current inhibition by Oxo-M (**Fig 1**; [20, 21]), documenting that OPC’s actions are not some unusual nonspecific effect. Lastly, OPC minimizes Ca^2+^ current inhibition by Oxo-M in PFC pyramidal neurons, extending the potential importance of PLA_2_ in M_1_R signaling to central neurons (**S1 Fig**).

Knockout mouse studies provide genetic evidence that N-current inhibition by M_1_R signaling requires cPLA_2_α by showing that cPLA_2_α^-/-^ SCG neurons exhibited little, while cPLA_2_α^+/+^ neurons exhibited robust N-current inhibition by Oxo-M (**Fig 3**). This interpretation of the data takes into account evidence that cPLA_2_α^-/-^ SCG neurons maintain normal functioning of all other components of the slow pathway. **First**, no significant differences between control N-current amplitude in cPLA_2_α^+/+^ vs cPLA_2_α^-/-^ SCG neurons were found, showing that N-channels exhibit normal activity **(Fig 3E)**. **Second**, AA inhibited whole-cell currents in cPLA_2_α^-/-^ neurons [19], demonstrating that channel sensitivity to a lipid signaling molecule downstream of cPLA_2_α remains unchanged. Complementary to these findings, imaging studies revealed that cPLA_2_α^-/-^ neurons release less fatty acid following exposure to Oxo-M, when compared to cPLA_2_α^+/+^ SCG neurons, indicating diminished downstream signaling [19]. **Third**, no significant differences between control M-current amplitude in cPLA_2_α^+/+^ vs cPLA_2_α^-/-^ SCG neurons were found, showing that M-channels exhibit normal activity **(Fig 4A)**. **Fourth**, M-current inhibition by Oxo-M remained robust in cPLA_2_α^-/-^ SCG neurons **(Fig 4)** with no significant change in the magnitude of M-current inhibition in cPLA_2_α^-/-^ SCG neurons, indicating no change in M_1_Rs, G_q_, or PLC, key players in the slow pathway. **Fifth**, the absence of cPLA_2_α appears to affect only specific aspects of N-current modulation by M_1_Rs with no effect on membrane-delimited M_2_/M_4_R signaling or M-current inhibition by M_1_R signaling. Alterations in unknown components in cPLA_2_α^-/-^ neurons might account for these changes rather than loss of cPLA_2_α. However, our Ab studies tested the consequences of an acute loss of cPLA_2_α activity in SCG neurons. Dialyzing wild-type neurons with cPLA_2_α Ab prevented N- but not M-inhibition by Oxo-M **(Fig 2G–2I)**, consistent with the knockout findings. Furthermore, Abs to sPLA_2_ had no effect on N-current, demonstrating a specific action of the cPLA_2_α Ab in blocking the slow pathway **(Fig 2B and 2C)**. These additional findings favor a model where N-current inhibition by M_1_Rs requires PIP_2_ breakdown by cPLA_2_α. In contrast, activated PLC is sufficient to cause M-current inhibition, revealing pathway divergence downstream of PLC with M- and N-current modulation by M_1_Rs.

PIP_2_ is thought to play a critical role in regulating Ca^2+^ channel activity where a bound PIP_2_ molecule facilitates coupling of voltage-sensing to channel opening [22, 24, 25, 28]. A requirement for cPLA_2_α is compatible with the idea that PIP_2_ bound to N-channels is selectively metabolized in situ by activated cPLA_2_α following M_1_R stimulation. Imaging studies document muscarinic stimulation of PIP_2_ breakdown occurring with a similar time course to Ca^2+^ current inhibition [24, 45]. The finding that antagonizing PLC blocks M- and N-current inhibition [21, 24, 36] complements these imaging studies and demonstrates that PLC activity is necessary for M_1_R modulation of both currents. However, these experiments do not provide direct evidence that rules out a requirement for cPLA_2_α during N-current modulation. Indeed, our studies document that preincubation with OPC, dialysis with a selective cPLA_2_α Ab, or the absence of cPLA_2_α, has no effect on M-current inhibition by M_1_Rs. These findings are consistent with a divergent signaling pathway where PIP_2_ dissociation from K^+^ channels, followed by breakdown by PLC, is necessary and sufficient for M-current inhibition. Minimal L- and N-current inhibition by Oxo-M occurred under these same conditions [19, 20] suggesting that PIP_2_ metabolism by PLC is insufficient for Ca^2+^ channel inhibition and consistent with a required downstream role for cPLA_2_α.

The requirement for additional phospholipases would protect N-channels from local Ca^2+^ influx chronically activating PLC to metabolize PIP_2_. Loss of PIP_2_ would stabilize channels in the closed state. These differences in M- versus N-current modulation by the slow pathway raise further intriguing questions about how different phospholipases might reside in channel microdomains or show varied affinities for different types of channels. These differences would impact which signaling molecules are generated locally to uniquely modulate N-channel activity. Recent evidence suggests that in addition to PIP_2_, other anionic phospholipids such as phosphatidylserine may bind specific sites on Ca^2+^ channel subunits that also may regulate and participate in channel modulation [46, 47]. From the same start point (e.g., a phospholipid molecule bound to an M- or N-channel), varied channel activity could occur as a result of phospholipid metabolism by different phospholipases. Thus, from moment to moment, differential phospholipase access to N-channels could give rise to varied current modulation by M_1_Rs with the potential to create highly localized synaptic plasticity.

Lastly, we examined cPLA_2_α’s effect on membrane excitability to test for its importance under more physiological conditions. In other SCG preparations, different subgroups of neurons were distinguished based on different AP firing patterns including *i*) firing of just one AP before adapting, *ii*) phasic firing of several AP before adapting and *iii*) tonic firing throughout the current injection pulse [48–49]. The vast majority of cPLA_2_α^+/+^ neurons exhibited phasic bursting as illustrated in **Figs 5A and 6A**. No cPLA_2_α^+/+^ neurons were tonically active and only 5/34 neurons fired single APs before adapting. Our studies unexpectedly revealed that the absence of cPLA_2_α dramatically increases AP firing in SCG neurons (**Figs 5 and 6**). These findings suggest that cPLA_2_α acts in a similar manner on N-channel activity in all types of mouse SCG neurons.

We interrogated the AP firing of phasic and tonically active neurons in more detail. A reduced latency to the first AP along with a shortened period of membrane repolarization following the AHP, and loss of spike adaptation can account for the increased AP frequency of firing. Resting membrane potential, threshold depolarization, AP amplitude, and AHP amplitude, and spike width did not change significantly **(Fig 6G–6J)**. In contrast, the absence of cPLA_2_α had little effect on increased firing following Oxo-M most likely because the firing rate already had reached maximal rates for SCG neurons, calculated to be 20–25 Hz [51]. Alternatively, increased SCG neuron firing that normally follows exposure to Oxo-M is attributed largely to inhibition of M-current [52], and we have shown that M-current is insensitive to the absence of cPLA_2_α consistent with the similar observed firing frequencies in cPLA_2_α^+/+^ and cPLA_2_α^-/-^ neurons (**Fig 6D and 6H**). Increased basal AP frequency in cPLA_2_α^-/-^ neurons suggests that tonic cPLA_2_α activity normally suppresses membrane excitability in wild-type neurons. Inward Ca^2+^ currents typically contribute to the duration of the AP overshoot; however, no obvious change occurred in the overshoot duration including the down slope of the AP **(Fig 6)**. Therefore, we suspect that in the absence of cPLA_2_α other channels sensitive to membrane PIP_2_ levels and/or its metabolites may exhibit altered tonic activity in cPLA_2_α^-/-^ neurons, whereas N-channels only become susceptible to the actions of cPLA_2_α following muscarinic stimulation.

A large number of channels expressed by SCG neurons exhibit sensitivity to phospholipids and their downstream metabolites. K^+^ channels determine the AP duration, frequency, and ability to fire repetitively [53]. In SCG neurons, a number of K^+^ currents control the length of the AHP including small Ca^2+^-activated K^+^ (SK3) currents [54, 55] and currents arising from the Kv2 channel family [56, 57]. Many of these types of K^+^ channels in SCG neurons bind PIP_2_ [58, 59] however, not all PIP_2_ sensitive K^+^ channels are affected by cPLA_2_α since we have shown that it has little effect on M-current. Some K^+^ channels, e.g., SK and BK channels are enhanced by both PIP_2_ and cPLA_2_α’s metabolite AA [60–63]; whereas other channel activity is enhanced by PIP_2_ but inhibited by AA [64]. Additionally PIP_2_ shifts HCN channel open probability versus test potential ~20 mV in the negative direction, increasing rates of neuronal firing [65, 66]. These channels are also present in SCG neurons [50] and therefore an increase in their activity may participate in increasing cell firing in cPLA_2_α^-/-^ SCG neurons. Lastly, free fatty acids, including AA, inhibit a number of different Na_V_ channels often by promoting inactivation (see N’Avanzo 2016 [67] for examples). Loss of AA release by cPLA_2_α from phospholipids would be predicted to lower Na_V_ channel inactivation and could underlie the long-lasting spiking that we observed in cPLA_2_α^-/-^ SCG neurons. These examples underscore how such varied changes in activity of multiple channel types make it virtually impossible to determine how many channel types in SCG neurons might be sensitive to cPLA_2_α activity and therefore exhibit gating changes in cPLA_2_α ^-/-^ SCG neurons.

## Conclusions

cPLA_2_α participates in various neurophysiological and neuropathophysiological events, including neurotransmitter release, long-term potentiation, membrane remodeling, neuronal death following cerebral ischemia, neurodegeneration and apoptosis [5, 6, 68, 69]. We have found at the cellular level, that cPLA_2_α participates in whole-cell N-current modulation by M_1_R signaling. Our findings that AP firing frequency increases with loss of cPLA_2_α expression reveals a role for tonic lipid processing in regulating membrane excitability that may underlie some of cPLA_2_α’s functions at the systems level. Thus, our findings identify two new roles for cPLA_2_α: mediating N-channel inhibition by M_1_R signaling and regulating membrane excitability.

## Supporting information

S1 Methods Method for dissociating rat prefrontal cortical neurons. Acutely dissociated pyramidal neurons from the prefrontal cortex (PFC) of young adult (2–4 weeks-old) Sprague–Dawley rats were obtained by removing the anterior aspect of the cortex following decapitation. Pieces were placed in DPBS at 4°C. PFC pieces were manually dissected into smaller pieces with a scalpel blade, and digested with papain (2 mg/ml) (Sigma) in Neurobasal-A medium (Life Technologies) bubbled with a 95% O_2_/5% CO_2_ gas mixture at 37°C in a shaking water bath for 60 minutes. After enzyme treatment, tissues were washed with Neurobasal-A medium containing bovine serum albumin (1 mg/ml) (Sigma) and trypsin inhibitor (1 mg/ml) (Sigma). Tissues were transferred into Neurobasal-A medium supplemented with 20 μl/ml of B27 (Invitrogen), 10% fetal bovine serum, 0.5 mM glutamine, and penicillin (100 U/ml)-streptomycin (0.1 mg/ml). Cortical neurons were dissociated by gentle trituration with a fire-polished Pasteur pipette; the supernatants after trituration were collected and mixed. Dissociated PFC neurons were then plated onto poly-*L*-lysine-coated glass coverslips in 25 mm^2^ dishes and placed at 37°C in a CO_2_ (5%) humidified incubator. Cells were pretreated with PTX for at least 5 hours before recording.
(PDF)

S1 Fig OPC eliminates whole-cell Ca^2+^ current inhibition by Oxo-M recorded from dissociated, large pyramidal-shaped neurons. (**A)** Bath application of Oxo-M (10 μM) inhibits the whole-cell Ca^2+^ current from a PFC pyramidal neuron shown in the plot of current amplitude vs time (left) and in individual sweeps (right) taken from the time course. (**B)** In contrast, OPC (10 μM) blocked current inhibition shown in the plot of current amplitude vs time (left) and in the individual sweeps (right) taken from the time course.
(PDF)

S2 Fig Schematic of how the different aspects of APs, presented in Fig 6C–6J, were measured. Spike Width, duration of AP at V½ of the AP amplitude. Rising Phase, time from V^1/^3 of AP to peak of AP. Falling Phase, time from AP peak to V^1/^3 of AP. Peak to Trough, time from AP peak to AHP.
(PDF)
