# Supplementary material for: cPLA2α-/- sympathetic neurons exhibit increased membrane excitability and loss of N-Type Ca2+ current inhibition by M1 muscarinic receptor signaling
Source: PLoS One. 2018 Dec 17;13(12):e0201322. doi: 10.1371/journal.pone.0201322 (PMC6296557; doi:10.1371/journal.pone.0201322)
Supplement: S1 Methods — Acutely dissociated pyramidal neurons from the prefrontal cortex (PFC) of young adult (2–4 weeks-old) Sprague–Dawley rats were obtained by removing the anterior aspect of the cortex following decapitation. Pieces were placed in DPBS at 4°C. PFC pieces were manually dissected into smaller pieces with a scalpel blade, and digested with papain (2 mg/ml) (Sigma) in Neurobasal-A medium (Life Technologies) bubbled with a 95% O2/5% CO2 gas mixture at 37°C in a shaking water bath for 60 minutes. After enzyme treatment, tissues were washed with Neurobasal-A medium containing bovine serum albumin (1 mg/ml) (Sigma) and trypsin inhibitor (1 mg/ml) (Sigma). Tissues were transferred into Neurobasal-A medium supplemented with 20 μl/ml of B27 (Invitrogen), 10% fetal bovine serum, 0.5 mM glutamine, and penicillin (100 U/ml)-streptomycin (0.1 mg/ml). Cortical neurons were dissociated by gentle trituration with a fire-polished Pasteur pipette; the supernatants after trituration were collected and mixed. Dissociated PFC neurons were then plated onto poly-L-lysine-coated glass coverslips in 25 mm2 dishes and placed at 37°C in a CO2 (5%) humidified incubator. Cells were pretreated with PTX for at least 5 hours before recording. (PDF) [file pone.0201322.s001.pdf]

Supporting Information

$cPLA_2\alpha^{-/-}$ Sympathetic Neurons Exhibit Increased Membrane Excitability and Loss of N-Type Ca^{2+} Current Inhibition by M_1 Muscarinic Receptor Signaling

Liwang Liu, Joseph V. Bonventre, and Ann R. Rittenhouse

S1 Fig Methods. Method for dissociating rat prefrontal cortical neurons. Acutely dissociated pyramidal neurons from the prefrontal cortex (PFC) of young adult (2-4 weeks-old) Sprague–Dawley rats were obtained by removing the anterior aspect of the cortex following decapitation. Pieces were placed in DPBS at 4°C. PFC pieces were manually dissected into smaller pieces with a scalpel blade, and digested with papain (2 mg/ml) (Sigma) in Neurobasal-A medium (Life Technologies) bubbled with a 95% O_2/5% CO_2 gas mixture at 37°C in a shaking water bath for 60 minutes. After enzyme treatment, tissues were washed with Neurobasal-A medium containing bovine serum albumin (1 mg/ml) (Sigma) and trypsin inhibitor (1 mg/ml) (Sigma). Tissues were transferred into Neurobasal-A medium supplemented with 20 µl/ml of B27 (Invitrogen), 10% fetal bovine serum, 0.5 mM glutamine, and penicillin (100 U/ml)-streptomycin (0.1 mg/ml). Cortical neurons were dissociated by gentle trituration with a fire-polished Pasteur pipette; the supernatants after trituration were collected and mixed. Dissociated PFC neurons were then plated onto poly-lysine-coated glass coverslips in 25 mm^2 dishes and placed at 37°C in a CO_2 (5%) humidified incubator. Cells were pretreated with PTX for at least 5 hours before recording.
