# Supplementary material for: cPLA2α-/- sympathetic neurons exhibit increased membrane excitability and loss of N-Type Ca2+ current inhibition by M1 muscarinic receptor signaling
Source: PLoS One. 2018 Dec 17;13(12):e0201322. doi: 10.1371/journal.pone.0201322 (PMC6296557; doi:10.1371/journal.pone.0201322)
Supplement: S1 Fig — (A) Bath application of Oxo-M (10 μM) inhibits the whole-cell Ca2+ current from a PFC pyramidal neuron shown in the plot of current amplitude vs time (left) and in individual sweeps (right) taken from the time course. (B) In contrast, OPC (10 μM) blocked current inhibition shown in the plot of current amplitude vs time (left) and in the individual sweeps (right) taken from the time course. (PDF) [file pone.0201322.s002.pdf]

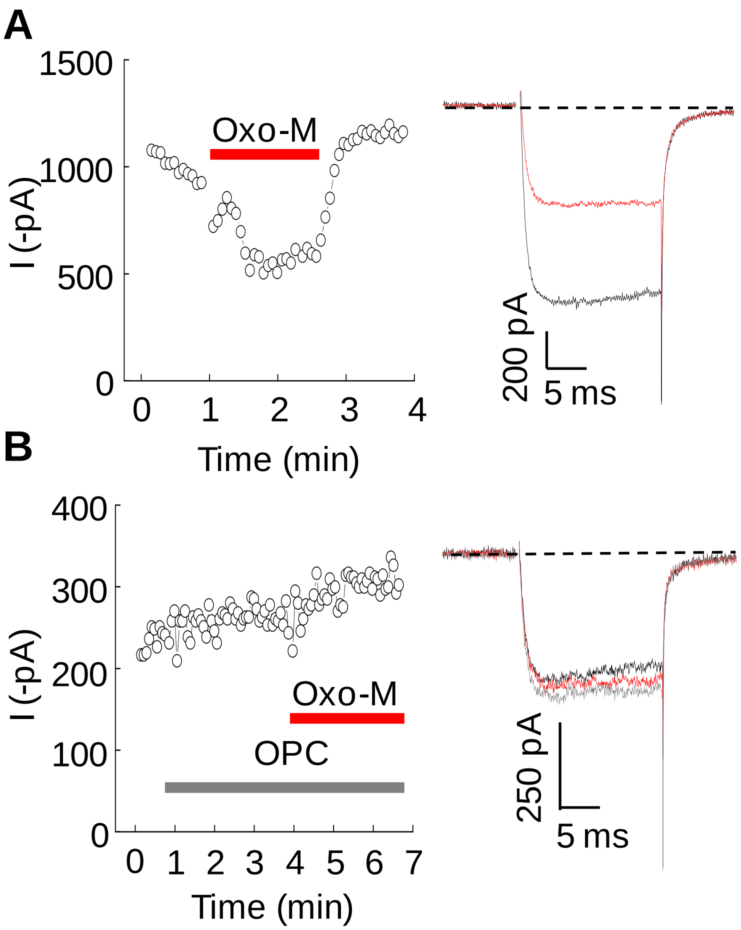

S1 Fig. OPC eliminates whole-cell Ca^{2+} current inhibition by Oxo-M recorded from dissociated, large pyramidal-shaped neurons. (**A)** Bath application of Oxo-M (10 µM) inhibits the whole-cell Ca^{2+} current from a PFC pyramidal neuron shown in the plot of current amplitude vs time (left) and in individual sweeps (right) taken from the time course. (**B)** In contrast, OPC (10 µM) blocked current inhibition shown in the plot of current amplitude vs time (left) and in the individual sweeps (right) taken from the time course.
