# Supplementary material for: cPLA2α-/- sympathetic neurons exhibit increased membrane excitability and loss of N-Type Ca2+ current inhibition by M1 muscarinic receptor signaling
Source: PLoS One. 2018 Dec 17;13(12):e0201322. doi: 10.1371/journal.pone.0201322 (PMC6296557; doi:10.1371/journal.pone.0201322)
Supplement: S2 Fig — Spike Width, duration of AP at V½ of the AP amplitude. Rising Phase, time from V1/3 of AP to peak of AP. Falling Phase, time from AP peak to V1/3 of AP. Peak to Trough, time from AP peak to AHP. (PDF) [file pone.0201322.s003.pdf]

Supporting Information

cPLA$_2$α$^{-/-}$ Sympathetic Neurons Exhibit Increased Membrane Excitability and Loss of N-Type Ca^{2+} Current Inhibition by M_1 Muscarinic Receptor Signaling

Liwang Liu, Joseph V. Bonventre, and Ann R. Rittenhouse

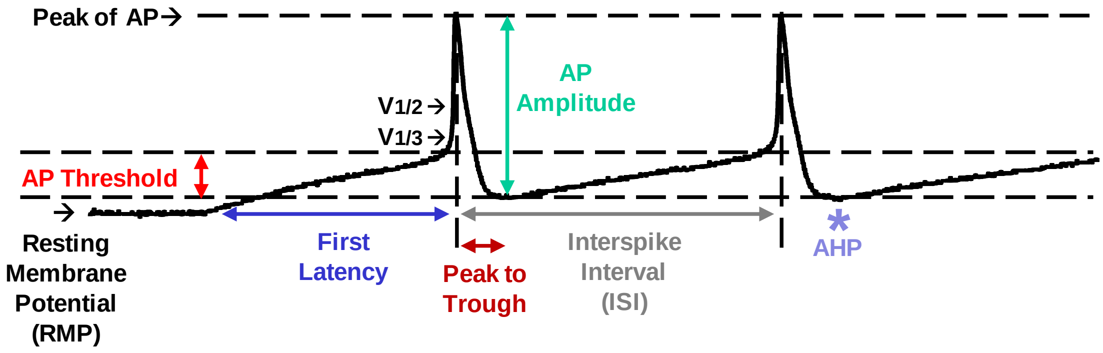

S2 Fig. Schematic of how the different aspects of APs, presented in Figs. 6C-J, were measured. Spike Width, duration of AP at V½ of the AP amplitude. Rising Phase, time from V$^{1/3}$ of AP to peak of AP. Falling Phase, time from AP peak to V$^{1/3}$ of AP. Peak to Trough, time from AP peak to AHP.
